# The Student’s Hand-Book of Surgical Operations

**Published:** 1904-10

**Authors:** 


THE STUDENT'S HAND-BOOK OF SURGICAL OPERATIONS. By Sir Frederick Treves (Bart.). New edition. 12mo. Illustrated. Limp cloth, $2.50 net. W. T. Keener & Co., Chicago.

This is a new abridged edition of Sir Frederick Treves' well-known manual of operative surgery, revised by the author and Jonathan Hutchinson, Jr., F. R. C. S., Surgeon to London Hospital and Examiner in Surgery, Royal Army Medical Department. It is illustrated with 121 cuts; 482 pages. It is an exhaustive work and makes things as plain as day to the beginner. I advise students and young graduates to buy it. D.

